# Participatory design of teacher dashboards: navigating the tension between teacher input and theories on teacher professional vision

**DOI:** 10.3389/frai.2023.1039739

**Published:** 2023-05-25

**Authors:** Anouschka van Leeuwen, Sebastian Strauß, Nikol Rummel

**Affiliations:** ^1^Department of Education, Utrecht University, Utrecht, Netherlands; ^2^Institute for Educational Research, Ruhr University Bochum, Bochum, Germany

**Keywords:** human centered design, participatory design, teacher dashboards, teacher professional vision, diagnostic judgements

## Abstract

In the field of AI in education, there is a movement toward human-centered design in which the primary stakeholders are collaborators in establishing the design and functionality of the AI system (participatory design). Several authors have noted that there is a potential tension in participatory design between involving stakeholders and, thus, increasing uptake of the system on the one hand, and the use of educational theory on the other hand. The goal of the present perspective article is to unpack this tension in more detail, focusing on the example of teacher dashboards. Our contribution to theory is to show that insights from the research field of teacher professional vision can help explain why stakeholder involvement may lead to tension. In particular, we discuss that the sources of information that teachers use in their professional vision, and which data sources could be included on dashboards, might differ with respect to whether they actually relate to student learning or not. Using this difference as a starting point for participatory design could help navigate the aforementioned tension. Subsequently, we describe several implications for practice and research that could help move the field of human centered design further.

## 1. Introduction

Following Luckin et al. (2016), we define AI as “computer systems that have been designed to interact with the world through capabilities and intelligent behaviors that we would think of as essentially human” (p. 14). Any AI application has to go through a full cycle of design, development, and deployment (De Silva and Alahakoon, 2022). Recently in what has been called the third wave of AI (Xu, 2019), the importance of human-centered AI has been recognized. This means that the envisioned goal for AI is to enhance human functioning rather than replacing it. To achieve this vision, it is imperative that the users of AI systems (i.e., stakeholders), are already involved in the design phase of any AI application. Involving stakeholders could lead to trustworthy AI solutions that have high interpretability, explainability, and robustness (De Silva and Alahakoon, 2022). Human-centered design is in line with the ideas underlying the research methodology Design-based Research (DBR). One of the pillars of DBR is joint ownership of the design by practitioners and researchers, thereby aiming to achieve alignment between practical needs of stakeholders and best practices derived from educational sciences (Kali et al., 2018; Hoadley and Campos, 2022).

For the process of designing human-centered technology, Dimitriadis et al. (2021) propose three principles for human-centered design, namely (1) agentic positioning of central stakeholders, (2) using a structured approach for the design phase, and (3) using educational theories to guide the whole process. While in some cases all three principles can indeed be applied to lead to effective solutions, in other cases the principles can also create tension. Dimitriadis et al. (2021) are also aware of this tension, noting that the principles can be at odds with each other when stakeholders are involved “without intimate knowledge of educational theories” (p. 286). Thus, a potential tension arises when input from stakeholders leads to different insights on the design of an AI system than input from educational theories does.

In this article, we provide an example of the tension between stakeholder involvement and educational theory for AI systems in education, namely *teacher dashboards*. Teacher dashboards are visual displays that capture and visualize student activities, with the aim to inform teachers about what is happening in their classroom (Van Leeuwen et al., 2022). The underlying idea is that the dashboard enhances the teacher's practice by complementing or augmenting the teacher's capabilities: the dashboard provides continuously captured and analyzed data that the teacher in turn can interpret and use for instructional decision making (Holstein et al., 2020). For example, when teachers support groups of collaborating students, the multitude of student activity at individual and group level can be overwhelming to monitor (Van Leeuwen et al., 2015). Teacher dashboards can provide an overview by aggregating information, and offering information about aspects of collaboration that are difficult to monitor. A compelling analogy is that dashboards support teachers “so that they no longer need to “drive blind” ” (Duval, 2011, p. 9). For more insights about teacher dashboards that go beyond the information necessary for the arguments presented below, see Van Leeuwen et al. (2022).

In line with the general trend in AI applications, the field of teacher dashboards has moved toward including the primary stakeholders - teachers - in the design phase. Including teachers this way, firstly, acknowledges the right of stakeholders to provide input on technology that directly relates to their practice (Sarmiento and Wise, 2022). Furthermore, teacher involvement is seen as an essential tool in countering the often low uptake of dashboards in practice (Kaliisa et al., 2022). However, as expressed above, teacher involvement may also lead to tension (Holstein et al., 2020). As Holstein et al. (2020) argue, “It may not always be desirable for AIEd systems to adapt to human facilitators' instructional goals… Teachers' … goals may be fundamentally at odds with known instructional best practices” (p. 5).

Several authors have noted this tension between stakeholder needs and insights from educational research. On the one hand, tension can arise when insights from educational research do not play a considerable part in the design process, and instead the stakeholders' input is leading (Luckin et al., 2013; Vezzoli et al., 2020). Vezzoli et al. (2020) for example described that teachers (stakeholders) did not adopt a critical stance toward the data indicators available to them, leading to a suboptimal design solution that in the end would not benefit student learning. On the other hand, tension can also arise when stakeholders do not feel seen or heard in their needs and wishes (Chatti et al., 2020). As a result, teachers may be reluctant to use the technology and the purpose of the design process has been defeated.

The goal of the present article is to zoom in on the tension between design principles and insights from educational research in the specific example of teacher dashboards to illustrate how the tension may be unpacked and turned into a productive situation. We will do so by relating insights from the field of teacher professional vision to participatory design of teacher dashboards. For both lines of work, a central element is teacher *vision* in the classroom and how to increase or optimize it.

In the remainder of this article, we first provide a brief description of participatory design of teacher dashboards and then move into relevant aspects of literature concerning teacher professional vision. Then, we will elaborate on the implications of connecting insights from the field of teacher professional vision to participatory design practices. We end by zooming out again and focusing on implications for human-centered design of AI systems in education.

## 2. Participatory design for teacher dashboards

There has been a movement toward including teachers as stakeholders in the design of dashboards (Dimitriadis et al., 2021). In their review of 90 participatory design papers, Sarmiento and Wise (2022) found that the stakeholders most often included were teachers (73%). Following Sarmiento and Wise (2022), we will use the term participatory design to refer to “processes of mutual learning in which designers collaborate with communities or stakeholders to inform the design of a technology they will use” (p. 535).

An important function of participatory design is to tailor the technological solution, in this case a teacher dashboard, to teachers' practices, perceptions and skills in a particular context. This agentic positioning of teachers in the design process is stressed by Dimitriadis et al. (2021) as the first principle of human-centered design. In participatory design, teachers often play a considerable role in establishing the design and functionality of the dashboard. In the review by Sarmiento and Wise (2022), 67% of research projects involved stakeholder input in early stages to identify the stakeholders' needs for the to be developed technology. Stakeholders are often asked about their practices and experiences in terms of what they notice in the classroom and how they subsequently act - in short, what information they rely on or which additional information they would like to incorporate in their instructional decisions. Technologies such as dashboards can be tailored to these individual needs by displaying specific information that the teachers in a particular context perceive as conducive to their monitoring of students' learning. Referring back to the earlier analogy of avoiding “driving blind”, the affordance of dashboards is that they offer possibilities for improving or expanding teacher vision in the classroom by providing teachers with more, or more accurate information. This in turn allows teachers to better tailor their support to their students' needs.

Thus, stakeholder involvement in case of teacher dashboards means that teachers are asked about the information they use in the classroom to tailor the AI system to that information. We will now regard how in this specific example tension may arise with educational theory. The leading educational theory on how teachers observe and monitor the classroom, which we will turn to now, is called *teacher professional vision*.

## 3. Teacher professional vision

Teacher professional vision is essential for successful teaching because it is the basis for knowing what is going on in the classroom. Professional vision consists of two steps (Van Es and Sherin, 2002): teachers first observe or *notice* certain behavior or characteristics in the classroom, and subsequently *interpret* those observations. Based on noticing and interpreting current events, teachers decide whether any pedagogical intervention is necessary, for example by providing support to a specific student.

The Diagnostic Judgements by Cognitive Modeling framework (DiaCoM) captures the factors that generally play a role in how teachers monitor a situation (Loibl et al., 2020). For the step of *noticing* or observing the classroom, teachers make use of sources of information in the classroom, also called *cues*. Cues can relate to the task students are working on, to the students themselves, or to aspects of the context in which learning occurs. Based on their interpretation of those cues, teachers arrive at a diagnostic judgment which forms the basis for deciding on further action in the classroom (e.g., providing additional help to particular students). Thus, it is vital which cues teachers pay attention to.

As explained in section 2, during participatory design, teachers are often asked about the sources of information they use or would like to use. Thus, in terms of the DiaCoM framework, participatory design often revolves around identifying the *cues* that teachers use, with the goal to design dashboards that capture those cues. Now that we have established this link, we will zoom in some more on cues and how teachers utilize them.

There are three aspects to be aware of when it comes to cues. Below, we address these three aspects and explain how they relate to participatory design. We thereby try to connect the vocabulary from the two fields, thus unpacking the potential tension between stakeholder involvement and using educational theories in human-centered design of technology.

The first important aspect of cue use is that research shows that teachers vary in what cues they seek to form their judgements (Seidel et al., 2021) and that teacher education helps teachers learn to notice relevant cues (König et al., 2022). This also applies to collaborating students and their teachers; Kaendler et al. (2016) for example showed that teachers differed in their ability to identify diagnostic cues during student collaboration, which improved after a training program. These research findings have implications for participatory design as well. If teachers are asked for input about the cues they use, there is a dependency on whether the teacher has already mastered the skill of noticing all relevant cues.

This is particularly relevant because the second important aspect of cue use is that there is a difference between diagnostic and non-diagnostic cues. Diagnostic cues are sources of information that pertain to student learning. In our earlier example of collaborating students, research shows that diagnostic cues include students asking reflective questions to each other and providing explanations for one's reasoning, because these behaviors relate to student learning (Meier et al., 2007). It is sometimes argued that a diagnostic cue also includes whether students show equal participation in the group discussion (Hrastinski, 2008). However, the *frequency* of *amount* of participation is not diagnostic; far more important is the *quality* of a student's contribution (Hrastinski, 2008; Strauß and Rummel, 2021). Group members who make fewer or shorter contributions may therefore not automatically be in need of support, and this cue is therefore not the best cue to act on for teachers.

If teachers monitor and act on diagnostic cues, there is a higher likelihood that the teacher's actions will eventually help to increase learning (Van Leeuwen and Janssen, 2019). Acting on non-diagnostic cues, on the other hand, could mean that the teacher spends valuable attention on aspects of behavior or the context that will not help to increase learning. Again, there are implications for participatory design because teachers may be asked to provide input on the kind of cues that are later integrated into the dashboard. If teachers report diagnostic cues, the design of the dashboard will indeed be improved. On the other hand, if teachers in their practice are used to monitoring non-diagnostic cues, the dashboard would consequently include these non-diagnostic cues as well. In this case, the final design of the dashboard may not be beneficial for teachers to use, and it may not be the best option to adapt the design of the dashboard exclusively to the teacher's practice.

The third and final aspect is that teachers not only need to notice cues but also *interpret* them (Van Es and Sherin, 2002). The resulting diagnosis of the situation can differ in whether it is accurate or not (Loibl et al., 2020). An accurate judgment means that the inference the teacher makes corresponds to an objective evaluation of the student, for example whether a teacher estimated a student's mathematical ability on the same level as a valid test would show. Accurate judgements are assumed to lead to decisions in the classroom that are more appropriate to the needs of the students. Inaccurate judgments are assumed to be detrimental to student learning because, as a result, the teacher is likely to provide students with instructional support that does not meet their current needs. For example, in Van Leeuwen et al. (2014) teachers were offered information whether collaborating groups showed agreement or critical debate in their conversation, based on research that indicates that critical yet constructive discussion is beneficial for learning (Weinberger and Fischer, 2006). Some teachers interpreted the occurrence of agreement as positive, because it showed them that the atmosphere in the group was good. Other teachers valued the role of constructive debate for learning. So, based on the same cue, teachers would arrive at different conclusions about whether a group was collaborating adequately, leading to different decisions for student support.

For participatory design, it means that even if teachers mention relevant, diagnostic cues and these cues are input for the design of the dashboard, it does not automatically mean that teachers will be able to correctly interpret the information on the dashboard. Instead, they may arrive at inaccurate conclusions that lead to suboptimal decisions about what support to offer to students.

## 4. Implications for the design of teacher dashboards

To summarize our line of reasoning so far, in the sections above we regarded a specific application of AI in education, namely teacher dashboards. To illustrate the relation between stakeholder involvement and educational theory, we have described that there is a close connection between participatory design of teacher dashboards, pedagogical content theories that describe the relevance of various cues teachers may use, and the research field of teacher professional vision that explains how teachers use cues and what the potential consequences are. Participatory design often involves a close examination of teachers' practice, including the cues teachers use or would like to use, with the aim to provide teachers with that information thus expanding their “vision” in the classroom. Pedagogical content theories, in turn, inform us about the relevance of cues in terms of whether or not they relate to student learning. And finally, research into teachers' professional vision aims at understanding the mechanics and boundaries of teacher professional vision in terms of the cues teachers use and how they interpret them. Professional vision gives us the vocabulary to describe what is happening in the classroom when teachers use dashboards.

Given these views, we can now articulate a new perspective on the tension between stakeholder involvement and educational theory for the specific case of teacher dashboards. When designing educational technology such as a teacher dashboard, we need to find the balance between acknowledging teachers' perspectives and needs and results from educational research on diagnostic cues. This includes incorporating the cues preferred by teachers, which is likely to increase the dashboard's usability and adoption, as well as the cues that lead to accurate diagnostic assessments. If the balance tips too far, tension can arise between tailoring a technological design to stakeholder needs and insights from educational theory. Tension in this case means that the usefulness (i.e., promoting pedagogically desired outcomes) of the dashboard may differ considerably depending on the balance between input from stakeholders and from educational theory.

These two views do not necessarily contradict each other. In line with human-centered design, we argue that participatory design needs to take into account teachers' *full* practice, including the mechanics of teacher professional vision and the strengths and potential boundaries thereof. Below, we provide two implications of our investigation that could turn the tension in this specific example into a productive situation. In section 5, we zoom out and regard the overall relation between stakeholder involvement and educational theory.

### 4.1. Co-development of diagnostic cues

The first implication for human-centered design of teacher dashboards is that we advocate a distribution of roles during the co-design process. Teachers, with their professional knowledge regarding teaching and supporting students' learning, may steer the design process by helping identify the context for the dashboard and potential goals. Further, they can describe and explain the way they diagnose students' learning process in terms of cues they use for decision making. Researchers, on the other hand, may steer the process by providing knowledge on learning theories to inform and decide on the final selection of metrics based on the cues mentioned by teachers, which will be included on the dashboard (Dimitriadis et al., 2021). This also includes investigating what is known about diagnostic cues in the context in which the dashboard will be implemented.

Thus, a vital topic to discuss concerns whether the cues and metrics that researchers and teachers propose during the design process are diagnostic or not. One way to determine whether a cue is diagnostic is to study existing research literature. In addition, we propose that the design process should also include conducting empirical investigations into whether the metrics in the dashboard are predictive of the desired learning outcomes and whether these cues support teachers' planning of lessons or interventions in the classroom in order to create desirable outcomes for the students.

The input of the two partners may in part be determined by the teachers' level of experience and quality of professional vision. The choice for a particular teacher or group of teachers that are selected for participatory design is therefore vital to consider carefully and to be transparent about in research reports, something which is currently often not the case (Sarmiento and Wise, 2022). An implication for research is that it is important to have an instrument to measure teacher professional vision. Because teachers' knowledge is often context-specific, developing such instruments can be a challenge (Van Es and Sherin, 2002; Kaendler et al., 2016). While assessment of professional vision is currently mostly used as a starting point for further teacher learning, in the context of teacher dashboards it can also be valuable during the co-design process. For example, it could help to determine the type of dashboard a teacher could benefit from most (see section 4.2), and help to identify which teachers to invite for the participatory design process.

### 4.2. Preparing teachers to leverage digital diagnostic cues

In our example, we have mainly focused on the very first step of teacher professional vision: noticing cues. After this initial step, interpretation and action follow as well. There are thus three different steps, each requiring specific teacher competencies (Van Es and Sherin, 2002; Van Leeuwen and Rummel, 2022). The idea behind most teacher dashboards is that they provide cues for teachers to monitor, and thus tie in to and support the initial process of noticing. However, that does not automatically mean that teachers will also be able to accurately interpret the cues on the dashboard nor that they will be able to select relevant follow-up action. Therefore, once the dashboard is finalized, the process of implementing a dashboard may involve a phase of teacher professional development on how to incorporate the dashboard into the teachers' practice so that it complements their professional vision. The dashboard itself may also play a role in this process. With respect to the functionality of the dashboard one can distinguish between mirroring and advising dashboards. While mirroring dashboards only provide information (i.e., support to notice cues), advising dashboards may also aid by providing suggestions for how to interpret that information (i.e., support interpretation). For example, the tool developed by Berland et al. (2015) provides information – or cues – about students' progression on the task, *and* subsequently provides advice on how to interpret the information and on which students to pair for collaborative work. Further research could focus on how and when to use what type of dashboard (Van Leeuwen and Rummel, 2019).

## 5. General implications and conclusion

To conclude, there is consensus that participatory design is necessary to ensure equity and uptake of AI applications in education. At the same time, there is a need to include insights from educational theories in the design process. In this article we looked at the role of participatory design and educational theory in the example case of teacher dashboards, and how these two forms of input may lead to tension in the design process. Similar types of tension may arise for other AI systems when other stakeholders are involved (for example students or policy makers) and other educational theories are relevant (for example theories on student self-regulation or theories of educational innovation). The purpose of this article was to illustrate how to investigate the role of stakeholders and educational theory in more detail, and how to try to resolve the potential tension.

What can we learn from our example for the broader range of AI applications in education? The most important conclusion is that for human-centered design it is important to clearly establish the role of the input from stakeholders and theory. Questions that may guide this process are what is the stakeholders' level of expertise, and how much is known from research and educational theory about the functionality that the AI system should implement. In our example that was a wealth of research that could inform the participatory design process. In general, we therefore propose to make use of the best of both worlds by using existing research as a productive working ground for participatory designs. Human-centered design means tying in to stakeholders' practice by understanding that practice *fully*; which also means acknowledging the strengths and boundaries of that practice. By being aware of this during participatory design, the resulting AI system can be tailored to support the stakeholder fully. In the end, the benefit of building on insights from both practice and research, besides uptake and equity, is that systems could be developed that are effective in achieving their intended goal.

The ideas presented in this article (such as openly discussing stakeholder expertise or the value of educational theory) assume a collaboration between stakeholders and researchers that may not be self-evident. Effort is needed to develop a close collaboration and trusting relationship between stakeholders and researchers, especially when the earlier described tension arises. There is a wealth of research, including the insights gained via the research methodology of Design-based Research (Kali et al., 2018; Hoadley and Campos, 2022), that have described ways to set up effective research-practice partnerships (see for example the recent special issue edited by Goldman et al., 2021). As Tabak (2022) puts it, it is vital to achieve “a climate in which interactions operate on a level plane and each participant's perspective is invited and valued, but open to face-saving modifications” (p. 171). One way to do so is to approach the design process as a learning experience for both parties, so not only for the stakeholders, but also for the research team. Another potential way to strengthen the collaboration between stakeholders and researchers is to embed dashboard creation and dashboard use into teacher training programs. Teacher training programs are already concerned with the diagnostic value of cues by teaching what kind of student behavior to monitor in the classroom (König et al., 2022). The crucial role of teacher training is confirmed by the study by Murtonen et al. (2022), who discovered that pedagogically trained teachers were better able to detect critical events than non-trained teachers. A logical extension could therefore be to also include cue use in the context of technology in teacher training programs. Likewise, teacher training programs could set the basis for creating teacher-researcher partnerships.

Identifying the tension that may arise in human-centered design is the first step in developing technologies that augment human capabilities in order to support them in their professional practice. We hope to have shown an example of how these tensions can be solved through collaboration between research and practice that focuses on leveraging the strengths of both sides.

## Data availability statement

The original contributions presented in the study are included in the article/supplementary material, further inquiries can be directed to the corresponding author.

## Author contributions

AL was responsible for the conception of this work and drafting the first version of the article. SS and NR were responsible for drafting the first version of the article and critical revision of the article. All authors contributed to the article and approved the submitted version.
